# 5-Fluorouracil Neurotoxicity in a Patient With Normal Dihydropyrimidine Dehydrogenase Activity

**DOI:** 10.7759/cureus.49898

**Published:** 2023-12-04

**Authors:** Ulaganathan Natarajan, Afoma Onyechi, Jessica Ohemeng-Dapaah

**Affiliations:** 1 Internal Medicine, SSM Health St. Mary's Hospital, St. Louis, USA

**Keywords:** 5-fu neurotoxicity, 5 fluorouracil, 5-fu toxicity, dihydropyrimidine dehydrogenase deficiency, uridine triacetate

## Abstract

5-fluorouracil (5-FU) is a well-known chemotherapeutic agent used for the treatment of colon cancer and other solid malignancies. Dihydropyrimidine dehydrogenase (DPD) is an enzyme that catalyzes 5-FU, and if a patient is deficient, such as through a gene mutation, they can be predisposed to severe toxicity. Although 5-FU-induced neurotoxicity is extremely rare, it can be fatal. We report a case of 5-FU neurotoxicity in a 56-year-old male patient with keratinizing squamous cell carcinoma of the anal canal on concurrent chemoradiation therapy consisting of 5-FU, mitomycin, and radiotherapy. Encephalopathy, dysarthria, and ataxia were noted on day three of treatment. MRI of the brain showed a pattern of global anoxic brain injury. DPD testing was negative for polymorphism, and the patient’s symptoms improved after treatment with uridine triacetate, the treatment for 5-FU toxicity.

## Introduction

5-fluorouracil (5-FU) is an antineoplastic agent used in the treatment of various malignancies such as colorectal, breast, stomach, and pancreatic cancer. When 5-FU is given as a single agent for chemotherapy, the primary mechanism of its cytotoxicity is by inhibiting cellular thymidylate synthase (TS) and integration of its metabolites into ribonucleic acid (RNA), thereby preventing deoxyribonucleic acid (DNA) replication and synthesis of RNA. When combined with other chemotherapeutics, its mechanism of action is unclear [1]. Common side effects of 5-FU include mucositis, myelosuppression, and gastrointestinal disturbances, such as nausea, vomiting, and diarrhea. Although neurotoxicity due to 5-FU is rare, it has been reported to cause both acute and delayed neurologic abnormalities [2]. The antidote for 5-FU toxicity is uridine triacetate. It should be administered as early as possible (ideally within 96 hours of symptom onset) [3]. A repeat brain MRI after uridine completion often shows the resolution of prior abnormalities.

Dihydropyrimidine dehydrogenase (DPD) is an enzyme produced in the liver involved in 5-FU catabolism. The prevalence of DPD deficiency varies; however, about 35% of people are estimated to have partial DPD deficiency [4]. Administration of 5-FU in patients with a complete absence of the enzyme can be fatal and is contraindicated [5]. Despite this association, studies have not shown a significant clinical correlation to necessitate pretreatment screening [6]. This is confirmed by case reports of patients who have developed 5-FU-associated neurotoxicity in the absence of DPD deficiency, as was seen in our patient [7]. The clinical signs of acute cerebellar syndrome secondary to 5-FU toxicity, which included ataxia, dysarthria, dysmetria, and nystagmus, were first characterized by Riehl and Brown [8]. Herein, we report a case of 5-FU neurotoxicity in a patient without DPD deficiency who was on treatment for keratinizing squamous cell carcinoma of the anal canal/perianal region.

## Case presentation

**Figure 1:**
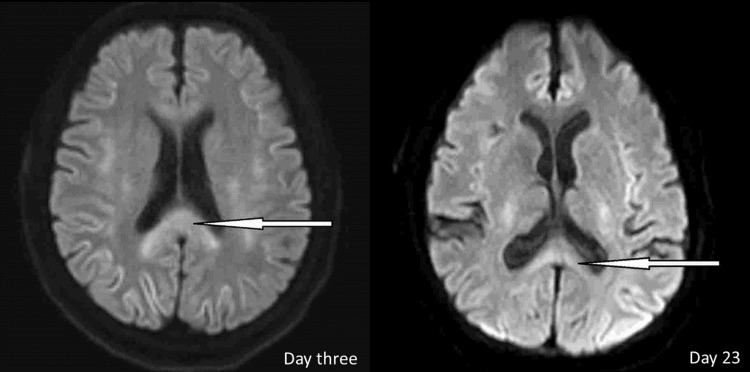
MRI of the brain obtained three days after symptom onset revealed significant diffusion restriction along the corpus callosum, which markedly improved on repeat MRI of the brain three weeks later

Our patient is a 56-year-old male with a past medical history of hypertension, chronic kidney disease (CKD) with baseline creatinine of 1.3 mg/dl, asymptomatic well-controlled HIV infection, and HPV infection. He was diagnosed with stage 3 moderate to poorly differentiated keratinizing squamous cell carcinoma of the anal canal/perianal region with no evidence of metastatic disease on staging scans. It was planned to start chemoradiotherapy with 5-FU at 1000 milligrams per square meters of body surface area infusion per day, days one to four and days 29-32, along with mitomycin 10 mg/m² to maximal 20 mg per dose on day one and day 29 along with concurrent radiation therapy. On day three of cycle one chemoradiation, the patient developed fatigue, nausea, vomiting, and poor appetite with poor oral intake. On day five, he was brought to the emergency room by a family member with altered mental status and an inability to walk. In the emergency room, the patient was ill-appearing, alert, oriented only to self, and had diffuse hyporeflexia. Labs showed elevated BUN of 52 mg/dl, creatinine of 2.36 mg/dl, and normal ammonia levels. He was evaluated with a CT head-per-stroke protocol, which was negative for bleeding, and an MRI brain was planned. He also received fluids with minimal improvement in mentation. Working differentials at that time were acute metabolic encephalopathy from marked volume depletion and cerebrovascular accident. On day six of cycle one (three days since symptom onset), the patient was awake, alert, and oriented to self and place but did not know the date or day, had difficulty with complex calculations and coordination. He also had ataxia for the left finger-nose-finger and was unable to have his gait tested because he felt weak. The prerenal azotemia improved with creatinine normalizing to his baseline after he received intravenous fluids. MRI of the brain (Figure 1) revealed diffusion restriction in a ribbon-like pattern along the cortex with some predominance of the posterior frontal and parieto-occipital region and significant diffusion restriction along the corpus callosum and middle cerebellar peduncle. Subtle changes in the T2 signal were also noted without any contrast enhancement. These findings are not consistent with stroke, hypoxic-ischemic injury, HIV encephalitis, or posterior reversible encephalopathy syndrome (PRES).

On day seven of the chemo cycle one (four days since symptom onset), the patient was able to perform three-step commands but still had slight speech difficulty, ataxic gait and needed assistance to ambulate. Oncology and neurology discussed and initiated treatment with uridine triacetate 10 mg per oral every six hours for 20 doses to counteract the neurotoxic effects of 5-FU. On day eight of the chemo cycle one (five days since symptom onset), the patient ambulated independently with improved speech. He remained inpatient to complete the uridine triacetate and was discharged home with no assistance. 

A repeat MRI of his brain (Figure 1) on day 26 of chemo cycle one (23 days since symptom onset) revealed significant interval improvement of bilaterally symmetric cytotoxic edema with the supratentorial and infratentorial compartments and mild residual involvement of the splenium of the corpus callosum.

## Discussion

5-FU neurotoxicity is common in patients with DPD deficiency but can occur in patients without DPD deficiency, as in our patient [9]. The cornerstone of a diagnosis of 5-FU neurotoxicity is the recent administration of the drug. The resulting encephalopathy should not be attributable to other metabolic causes such as stroke, non-convulsive epilepsy, infection, hepatic encephalopathy, or uremic encephalopathy and should not be ascribable to other medications [10]. Radiologic diagnosis is with contrast-enhanced MRI of the brain, with diffusion-weighted imaging. Characteristic findings are diffuse bilateral symmetrical hyperintensities in the T2W flair images with corresponding areas of diffusion restriction in periventricular deep white matter and corpus callosum [11].

Our patient started to show signs of improvement after stopping the 5-FU treatment, which was well-pronounced after the administration of uridine triacetate. In many cases, withdrawal of therapy alone results in the resolution of symptoms and corresponding normalization of MRI findings [12]. Uridine triacetate is a pyrimidine analog which, when administered, competes with 5-FU metabolites and hence serves as an antidote for severe toxicities caused by 5-FU and in cases of 5-FU overdose [13]. The best outcomes are when uridine triacetate is administered within 96 hours of 5-FU administration, but it can be administered after that time [14].

## Conclusions

In conclusion, clinicians should always suspect 5-FU-induced neurotoxicity in any patient on 5-FU-based chemotherapy presenting with acute, unexplained neurological symptoms. Prompt discontinuation of medication, alongside the administration of uridine triacetate, are vital steps in treatment. Although it has been postulated that DPD deficiency predisposes patients to 5-FU toxicity, patients with normal DPD activity and levels are also at increased risk of its adverse effects.
